# Effects of resistance exercise on patients with post-stroke dysphagia based on ACSM recommendations: a systematic review of randomized controlled trials

**DOI:** 10.3389/fphys.2025.1623298

**Published:** 2025-08-06

**Authors:** Yu Ye, Kairui Wu, Yingquan Liu, Hongjie Ji, Hongtao Li, Bo Jiang, Fangyuan Xu, Xuejun Li, Peijia Hu, Hongliang Cheng

**Affiliations:** ^1^ Graduate School of Anhui University of Chinese Medicine, Anhui, China; ^2^ Graduate School of Guangzhou University of Chinese Medicine, Guangdong, China; ^3^ The Second Affiliated Hospital of Anhui University of Chinese Medicine, Anhui, China

**Keywords:** post-stroke dysphagia, resistance exercise, ACSM recommendations, exercise dose, swallowing function, swallowing safety

## Abstract

**Background:**

Resistance exercise shows potential for improving swallowing function in post-stroke dysphagia (PSD), though optimal dose-response parameters remain unclear. While the American College of Sports Medicine (ACSM) framework effectively guides exercise prescriptions in healthy populations, its application to PSD rehabilitation lacks meta-analytical validation. This study evaluates varying resistance exercise dosages on swallowing outcomes in PSD patients.

**Methods:**

We systematically searched PubMed, Embase, Web of Science, and Cochrane for randomized controlled trials (RCTs) investigating resistance training in PSD. Interventions were stratified using ACSM compliance criteria (6/8 and 7/8 thresholds) into high-adherence versus low/uncertain-adherence groups. Subgroup analyses employed random-effects meta-analyses.

**Results:**

Analysis included 19 RCTs (n = 566). Using 6/8 ACSM criteria, 11 studies comprised the high-adherence group and eight the low/uncertain group. High-adherence interventions demonstrated improved positively oriented scores [SMD = −1.72 (95% CI −3.26 to −0.18)], enhanced safety [SMD = −0.93 (95% CI −1.54 to −0.32)], and worsened negatively oriented scores [SMD = 2.27 (95% CI 0.66–3.87)]. Low-adherence groups showed non-significant improvements in positively oriented scores [SMD = −0.47 (95% CI −1.02 to 0.09)], negatively oriented scores [SMD = 0.43 (95% CI −0.09–0.94)], and safety [MD = −1.85 (95% CI −3.83 to 0.13)]. Applying stricter 7/8 criteria reclassified nine studies as high-adherence and 10 as low/uncertain. High-adherence groups exhibited greater positively oriented scores improvement [SMD = −2.15 (95% CI −4.11 to −0.20)], safety enhancement [MD = −1.05 (95% CI −1.58 to −0.51)], and negatively oriented scores decline [SMD = 2.85 (95% CI 0.82–4.89)]. Low-adherence groups maintained non-significant outcomes: positively oriented scores [SMD = −0.33 (95% CI −0.75 to 0.10)], negatively oriented scores [SMD = 0.32 (95% CI −0.09–0.74)], and safety [MD = −1.39 (95% CI −2.84 to 0.07)].

**Conclusion:**

Resistance exercise demonstrates superior therapeutic effects over non-resistance interventions for PSD. High adherence to ACSM-recommended dosages yields significantly greater improvements in swallowing function and safety compared to low/uncertain adherence regimens. These findings validate the clinical utility of ACSM guidelines for optimizing resistance exercise prescriptions in PSD rehabilitation.

**Systematic Review Registration:**

https://www.crd.york.ac.uk/PROSPERO/, identifier CRD420251041450.

## 1 Introduction

Dysphagia is a clinical disorder characterized by impaired coordination of the oropharyngeal and esophageal muscles, resulting in the inability to safely and efficiently transport liquid or food boluses from the oral cavity to the stomach (Matsuo and Palmer, 2008). The swallowing process involves the coordinated action of over 25 pairs of muscles, including those of the jaw, lips, cheeks, soft palate, larynx, pharynx, and esophagus (Sasegbon and Hamdy, 2017). As a common complication of stroke, the pathogenesis of dysphagia primarily stems from damage to key neurological structures such as the cortical swallowing centers, corticobulbar tracts, medullary swallowing centers, and extrapyramidal system (Arnold et al., 2016; Cheng et al., 2022). Stroke has been established as the second leading cause of death globally, affecting approximately 13.7 million individuals annually (Maciejewska et al., 2024). Depending on assessment tools, the prevalence of dysphagia in stroke patients ranges from 19% to 80% (Meng et al., 2000; Martino et al., 2005; Takizawa et al., 2016), with over 75% of early-stage post-stroke dysphagia (PSD) patients exhibiting moderate to severe symptoms (Rommel and Hamdy, 2016), significantly compromising daily function and quality of life.

Currently, no clinically specific pharmacological agents exist for patients with PSD, with current therapeutic approaches primarily focusing on peripheral and central stimulation methods, swallowing muscle resistance training, and traditional Chinese acupuncture (Xu et al., 2023). However, both peripheral/central stimulation methods and acupuncture are limited by their reliance on specialized rehabilitation equipment and assistance from healthcare professionals, leading to suboptimal treatment adherence among PSD patients. In contrast, swallowing muscle resistance training has gained widespread acceptance in PSD management due to its unique advantages of convenience and cost-effectiveness, demonstrating significant clinical value (Cheng et al., 2021). As a critical non-pharmacological intervention, resistance training not only enhances quality of life in the general population but also promotes cardiovascular functional recovery and improves motor control of associated muscle groups in post-stroke individuals (Choi et al., 2020; Hanssen et al., 2022).

Studies have demonstrated that resistance exercise effectively restores the sensorimotor control system of swallowing by stimulating laryngeal, hyoid, and epiglottic movements to protect the airway and modulating the opening of the upper esophageal sphincter (UES) to facilitate bolus transfer into the esophagus (Lang, 2009). By enhancing muscular strength, range of motion, and neuromuscular coordination, resistance training targeting swallowing-related musculature improves oropharyngeal swallowing function and reduces the risk of aspiration (Pauloski, 2008). A systematic review by Mepani et al. (Liu et al., 2022) further corroborated that resistance exercise could significantly enhance swallowing safety, oral intake capacity, and psychological wellbeing in PSD patients. Another meta-analysis (Zhang et al., 2025) confirmed that resistance exercise could selectively activate the sternocleidomastoid muscle, thereby improving patient adherence to rehabilitation protocols, reducing aspiration and pneumonia incidence, and elevating quality of life. The above studies overlap in their assertion of resistance exercise as an indispensable component of PSD rehabilitation strategies.

Nevertheless, no consensus has been reached regarding the optimal dose-response parameters of resistance training in the management of PSD. A dose-response study by Brittany N. Krekeler et al. (Krekeler et al., 2021) revealed that dysphagia exercise programs emphasizing resistance training often yield more pronounced therapeutic effects when utilizing higher resistance with fewer repetitions. In a study comparing various intensities of lingual resistance exercise, Van den Steen et al. (2019) failed to demonstrate significant differences in outcomes among training levels at 60%, 80%, or 100% of the one-repetition maximum (1RM) over an 8-week intervention. As a core intervention variable in exercise therapy, the precise delineation of the dose-response relationship for exercise dosage is a prerequisite for optimizing neuromuscular adaptations and clinical outcomes (Peterson et al., 2005; Ohkawara et al., 2007; Radaelli et al., 2015; Schoenfeld et al., 2017).

The American College of Sports Medicine (ACSM), based on biomechanical and exercise physiological evidence, has systematically established a land-based exercise prescription paradigm for healthy populations in its authoritative guidelines, ACSM’s Guidelines for Exercise Testing and Prescription (11th Edition), encompassing resistance, cardiorespiratory, flexibility, and neuromotor training (Garber et al., 2011). Resistance exercise therapy administered to seemingly healthy adults in accordance with ACSM guidelines can modulate muscle hypertrophy and improve motor function (Rhea et al., 2003; Riebe et al., 2015), yet excessive exercise regimens yield negligible additional benefits. While studies on resistance exercise interventions for PSD have been increasing over the past few years, there remains a notable lack of research assessing exercise dosage based on ACSM guideline recommendations. Furthermore, it remains unclear whether ACSM exercise prescriptions are equally applicable to PSD populations and whether the recovery of swallowing function in PSD patients is positively correlated with adherence to ACSM guidelines during resistance exercise. Therefore, this review will categorize resistance exercise interventions into high-adherence versus low/uncertain-adherence groups based on their compliance with ACSM guideline recommendations and compare their intervention effects in PSD patients.

## 2 Materials and methods

The systematic literature review process was conducted strictly in accordance with the Preferred Reporting Items for Systematic Reviews and Meta-Analyses (PRISMA) statement and has been registered on the International Prospective Register of Systematic Reviews (PROSPERO) platform (CRD420251041450).

### 2.1 Search strategy

A systematic search was conducted across four electronic databases (PubMed, Embase, Web of Science, and Cochrane Library), encompassing records from database inception through 7 March 2025. The search strategy adhered to the Population, Intervention, Comparison, Outcomes, and Study Design (PICOS) framework, focusing on thematic relevance to resistance exercise interventions and associated clinical outcomes. The search primarily comprised the following combinations of Medical Subject Headings (MeSH) terms and keywords: (“Stroke” or “Strokes” or “Cerebrovascular Accident” or “cerebral infarction” or “Cerebrovascular Apoplexy” or “Brain Vascular Accident” or “Cerebrovascular Stroke” or “Apoplexy” or “CVA”) AND (“Deglutition Disorders” or “Deglutition Disorder” or “Dysphagia” or “Swallowing Disorders” or “Oropharyngeal Dysphagia” or “Dysphagia, Oropharyngeal” or “Esophageal Dysphagia”) OR (“post-stroke dysphagia” or “swallow dysfunction after stroke” or “dysphagia after stroke” or “swallowing disorder after dysphagia”) AND (“Exercise” or “Resistance Training” or “Exercise Movement Techniques” or “Exercise Therapy” or “Exercises” or “Training” or “resistance exercise” or “Physical Exercise” or “Acute Exercise” or “Exercise Program” or “Exercise Training” or “Physical Activity” or “Training, Resistance” or“Strength Training”). The comprehensive search strategy is outlined in Supplementary Appendix 1. To ensure comprehensive literature coverage, supplementary manual searches were performed through manual examination of reference lists in identified review articles. If necessary, their corresponding authors were contacted for further information.

### 2.2 Eligibility criteria

The study inclusion criteria were as follows: (a) Publicly published randomized controlled studies; (b) Adult subjects with a confirmed diagnosis of PSD; (c) Confirmation of PSD diagnosis by the Video Fluorescence Swallowing Study (VFSS) or Fibroendoscopic Swallowing Assessment (FESS) or the Standardized Swallowing Difficulty Assessment Instrument. (d) Experimental group interventions involving any type of resistance exercise. (e) Control groups receiving routine swallowing care (without resistance training) or no treatment; (f) Inclusion of swallowing function and/or swallowing safety as outcome indicators. There was no restriction on the language of publication.

The exclusion criteria for studies were as follows: (a) control interventions involving resistance exercise or non-standard treatments; (b) dysphagia not caused by stroke; (c) studies in which specific medications were administered during the resistance exercise intervention; (d) article types including reviews, case reports, meeting summaries, expert opinion articles, or peer-reviewed publications. (e) Republications of experimental studies; (f) Studies from which valid outcome data could not be extracted or were missing.

Two authors (WKR and YY) independently screened the article titles and abstracts. Full-text articles were reviewed if deemed potentially eligible by either author. An article was included if both authors agreed on its eligibility. Methodological discrepancies were resolved through iterative adjudication by the investigative team, with arbitration by the third and fourth authors (LYQ and JHJ) until scholarly consensus was achieved.

### 2.3 Data synthesis and analysis

Two investigators (YY and WKR) independently performed data extraction using standardized templates to capture the following domains: study metadata (authors, publication year, country), participant demographics (age, sex distribution, stroke types, side of stroke), exercise intervention parameters (modality, cycle frequency, repetitions, sets), control group protocols, and outcome measurement methodologies. For graphical data lacking textual quantification, Engauge Digitizer was implemented with pre-calibrated precision thresholds. Primary outcomes comprised swallowing function and swallowing safety, and functional living capacity (measured by the modified Barthel Index). All datasets underwent blinded duplicate verification by two corresponding authors (HPJ and CHL), with discrepancies resolved through third-party adjudication following predefined consensus protocols. The resistance exercise dosage in included studies was evaluated according to ACSM guidelines for enhancing muscle strength, range of motion, and swallowing function in PSD patients (Garber et al., 2011), with interventions classified into high-adherence versus low/uncertain-adherence groups based on ACSM recommendations. Two independent evaluators (YY and LYQ) systematically assessed exercise parameters (frequency, intensity, repetitions, and sets) to determine adherence levels, as detailed in Table 1.

**TABLE 1:** ACSM Recommendations for Resistance Exercise in healthy adults.

Exercise dose	Resistance exercise
Frequency	3–5 days/week, Training for the same muscle group takes at least 24 h
Intensity/workload	Start with 40%–50% of 1RM, no more than 60%–80% 1RM, 5–10 repetitions per group. Progressively increase the load (+2–10%), repetitions, or sets every 2–4 weeks to continuously stimulate adaptation
Duration	Group 2–5, intergroup rest can be extended to 2–3 min

Note: 1RM: one repetition maximum.

Each parameter’s minimum therapeutic dosage was assessed through a tripartite scoring matrix (2 = full compliance, 1 = uncertain compliance, 0 = non-compliance), where inter-rater discrepancies between independent evaluators YY and JHJ were reconciled via a structured adjudication process involving arbiter WKR. Among them, for intensity assessment, the core criterion was based on the ACSM recommendation that resistance exercise intensity should be defined as a percentage of one-repetition maximum (1RM) (60%–80% 1RM for adults) (Garber et al., 2011). When 1RM was not directly reported, surrogate indicators were used for judgment: (1) the Borg Rating of Perceived Exertion scale (the score of 12–15 corresponds to about 60%–80% 1RM); (2) for the study labeled “Ind.tail (individually tailored)” or “NR (not reported),” it is simply described as “gradually adjusted to the fatigue threshold based on patient tolerance,” lacking relevant information on resistance exercise intensity (neither 1RM percentage nor alternative indicator description), so it is judged as “uncertain compliance.” This framework enabled quantification of exercise programs’ compliance rates with ACSM guidelines across surveys. To ensure analytical robustness, dual classification thresholds were implemented. Initially, programs with ≥6/8 compliance were categorized as high adherence, and <6/8 as low/uncertain adherence. Subsequently, methodological refinement elevated the threshold to ≥7/8 compliance for high adherence classification, while studies below this cutoff were designated as low/uncertain adherence, thereby establishing standardized comparability across intervention cohorts.

### 2.4 Statistical analysis

Statistical analyses were conducted using Review Manager 5.4, with included studies stratified into two cohorts based on ACSM guideline adherence. Heterogeneity within subgroups was evaluated according to the Cochrane Handbook guidelines, incorporating the Higgins I^2^ statistics (Huedo-Medina et al., 2006). Effect sizes were calculated as mean difference (MD) or standardized mean difference (SMD) with 95% confidence intervals (95% CI). Statistical models were selected based on heterogeneity thresholds: fixed-effect models for I^2^ ≤ 50% and random-effects models for I^2^ > 50%. Publication bias was analyzed through funnel plots plotting effect sizes against standard errors across included studies.

### 2.5 Quality appraisal

Study quality was independently assessed by two author teams (CHL/HPJ and LHT/XFY) following Cochrane Collaboration guidelines for randomized controlled trial evaluation (Higgins et al., 2011), with the revised Cochrane Risk of Bias Tool (RoB 2) specifically designed for RCTs implemented in accordance with Cochrane Handbook recommendations (Sterne et al., 2019).

## 3 Results

### 3.1 Study identification and selection

**FIGURE 1:**
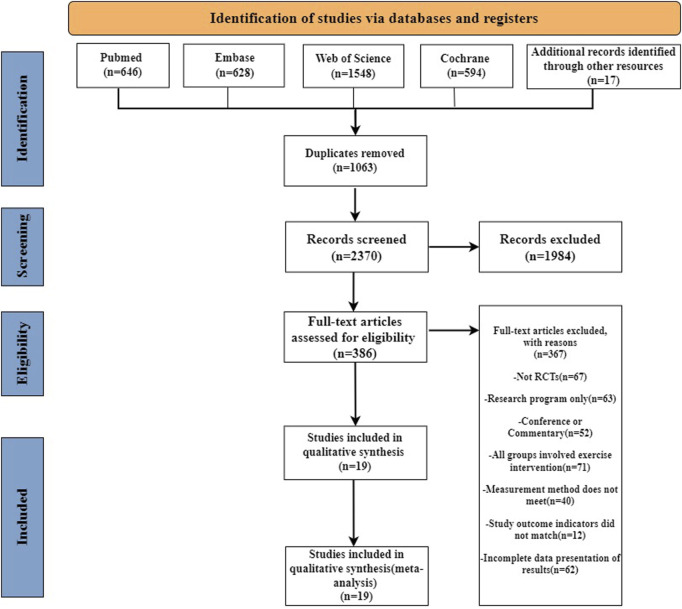
PRISMA study flow diagram.

Our systematic search identified 3,416 records across four databases: PubMed (n = 646), Embase (n = 628), Cochrane Library (n = 594), and Web of Science (n = 1,548), supplemented by 17 manually retrieved documents. Following de-duplication, 2,370 unique records underwent title/abstract screening, resulting in 386 articles progressing to full-text evaluation. Through rigorous full-text assessment, 19 eligible studies (Kang et al., 2012; Moon et al., 2016; Choi et al., 2017; don, 2017; Eom et al., 2017; Kim et al., 2017; Park et al., 2017; Moon et al., 2018; Park et al., 2018; Hwang et al., 2019; Jang et al., 2019; Kim and Park, 2019; Park et al., 2019; Arnold and Bausek, 2020; Carnaby et al., 2020; Park et al., 2020; Wang et al., 2022; Benfield et al., 2023; Liu et al., 2025) were ultimately included, with the complete selection process visually summarized in Figure 1.

### 3.2 Study characteristics

This systematic review included 19 studies involving a total of 566 PSD patients, with 283 participants allocated to the intervention group and 283 to the control group. The mean age of the intervention group ranged from 59.26 to 71.00 years, compared to 58.43–75.10 years in the control group. Regarding sex distribution, 55.48% of PSD patients in the intervention group were male (44.52% female), while the control group comprised 53.00% males and 47.00% females. Geographically, 14 studies originated from South Korea, with two studies each from China and the United States, and one study from the United Kingdom. Besides, 14 studies reported stroke types (e.g., ischemic vs. hemorrhagic), 10 studies detailed the affected hemisphere (e.g., left vs. right), and 17 studies clearly recorded the baseline data on the severity of swallowing function in PSD patients.

Regarding resistance exercise interventions, the intervention group implemented diverse protocols, including Effortful Swallowing Training (EST), Tongue-Pressure Resistance Training (TPRT), Tongue Strength and Accuracy Training (TSAT), Chin Tuck Against Resistance (CTAR), Head Lift Exercise (HLE), and Resistive Jaw Opening Exercise (RJOE). In contrast, the control group received conventional swallowing therapy without exercise components. The intervention duration varied from 2 to 8 weeks, with a frequency ranging from three sessions per week to twice daily, and session durations predominantly spanning 30–60 min. Regarding outcome function assessment, 17 studies evaluated swallowing function in patients with PSD, with 11 studies specifically assessed swallowing safety, as detailed in Table 2.

**TABLE 2:** Basic characteristics of research.

Author	Country	Year	Age (mean + SD)	Total/male/female	Stroke type (hemorrhage /infarction; n)	Side of stroke (Right/Left/both; n)	Severity of dysphagia (mean + SD)	Intervention	Control	Swallowing screening measures	Outcome
Kang JH et al.	Korea	2012	T:68.3(6.60) C:66.7(6.01)	T:25/16/9 C:25/18/7	T:10/15 C:8/17	NR	Clinical dysphagia Scale T:52.30(7.50) C:50.10(6.80)	pharyngeal, laryngeal, and respiratory exercises + CON Length of Intervention: 2 months Freq: daily Duration: 60 min	CON	VFSS、FOIS	Swallowing function, living ability
Choi JB et al.	Korea	2017	T:60.81(10.85) C: 60.40(10.50)	T:16/10/6 C:15/9/6	T:7/9 C:3/12	T:7/9/0 C:10/15/0	FOIS T:3.13(0.95) C:3.20(0.67)	Shaker exercise + CON Length of Intervention: 4 weeks Freq: 5 times a week Duration: 30 min	CON	FOIS	Swallowing function Swallowing safety
Eom MJ et al.	Korea	2017	T: 69.20(4.10) C: 70.20(3.60)	T:13/5/8 C:13/6/7	NR	NR	VDS T:57.96(9.39) C:55.04(9.38)	resistance EMST + CON Length of Intervention: 4 weeks Freq: 5 times a week Duration: 30–60 min	CON	VDS	Swallowing function Swallowing safety
Kim KY et al.	Korea	2017	T: 64.00(5.89) C: 61.80(4.79)	T:15/7/8 C:15/8/7	NR	T:10/5/0 C:7/8/0	VFSS T:43.73(9.80) C:41.56(8.56)	short neck flexion exercise + CON Length of Intervention: 6 weeks Freq: 3 times a week Duration: 30 min	CON + NMES	VFSS、ASHA-NOMS	Swallowing function
Robert J Arnold et al.	United States of America	2020	T:70.50(NR) C: 66.10(NR)	T:10/2/8 C:10/6/4	NR	NR	FOIS T:3.20(2.20) C:3.30(2.16)	resistance EMST + CON Length of Intervention: 4 weeks Freq: daily Duration: 15 min	CON	FOIS、MASA	Swallowing function Swallowing safety
Jang KW et al.	Korea	2019	T: 67.28(9.48) C: 71.15(8.61)	T:18/10/8 C:18/9/9	T:8/10 C:6/12	NR	FDS T:45.68(12.46) C:44.92(13.64)	MIE exercise + CON Length of Intervention: 2 weeks Freq: 5 times a week Duration: 30 min	CON	ASHA-NOMS, FDS	Swallowing function Swallowing safety
Park HS et al.	Korea	2019	T: 66.50(9.50) C: 64.80(11.20)	T:12/6/6 C:12/5/7	NR	NR	VDS T:61.79(9.12) C:59.58(9.56)	EST + CON Length of Intervention: 4 weeks Freq: 5 days a week, 2 times a day Duration: 30 min	CON	VDS	Swallowing function
Kim HD et al.	Korea	2017	T: 62.17(11.01) C: 59.29(10.19)	T:18/11/7 C:17/8/9	T:7/11 C:7/10	T:9/9/0 C:11/6/0	VDS T:37.36(11.09) C:42.67(13.71)	TPRT + CON Length of Intervention: 4 weeks Freq: 5 times a week Duration: NR	CON	VDS	Swallowing function Swallowing safety
Moon JH et al.	Korea	2016	T: 63.89(5.23) C: 64.78(4.99)	T:9/6/3 C:9/5/4	T:2/7 C:3/6	T:8/1/0 C:7/2/0	—	TSAT + CON Length of Intervention: 8 weeks Freq: 5 times a week Duration: 30 min	CON	\	Swallowing function
Moon JH et al.	Korea	2018	T: 62.00(4.17) C: 63.50(6.05)	T:8/3/5 C:8/4/4	T:2/6 C:2/6	NR	MASA T:145.50(3.38) C:144.13(5.46)	TPSAT + CON Length of Intervention: 8 weeks Freq: 5 times a week Duration: 30 min	CON	MASA	Swallowing function
Wang T et al.	China	2022	T: 60.22(8.83) C: 60.72(8.61)	T:18/10/8 C:18/11/7	T:8/10 C:9/9	T:10/8/0 C:11/7/0	—	TPRT + CON Length of Intervention: 4 weeks Freq: 5 times a week Duration: 20 min	CON	\	Swallowing safety
Kim HH et al.	Korea	2019	T:63.50(5.50) C: 65.20(6.20)	T:12/6/6 C:13/6/7	T:5/7 C:7/6	T:5/7/0 C:4/9/0	FOIS T:3.40(1.05) C:3.19(0.68)	Modified CTAR exercise + CON Length of Intervention: 6 weeks Freq: 5 times a week Duration: NR	CON	FOIS	Swallowing function Swallowing safety
Park JS et al.	Korea	2017	T: 59.26(11.94) C: 61.59(13.61)	T:13/9/4 C:14/8/6	T:8/5 C:7/7	NR	Kinematic of hyoid bone T:1.20(0.36) C:1.24(0.30)	HLE + CON Length of Intervention: 4 weeks Freq: 5 times a week Duration:<10 min	CON	Kinematic of hyoid bone, Kinematic of larynx	Swallowing safety
Benfield JK et al.	UK	2023	T:71.00 (10.40) C:75.1 (11.50)	T:12/6/6 C:15/2/13	T:2/10 C:4/11	NR	FOIS T:5.00(3.00) C:4.00(2.00)	swallow strength and skill training + CON Length of Intervention:2 weeks Freq: 5 times a week Duration: 45 min	CON	FOIS	Swallowing function Swallowing safety
Liu S et al.	China	2025	T:59.66(8.90) C:62.44(8.60)	T:29/21/8 C:29/21/8	NR	T:13/6/10 C:11/9/9	FOIS T:1.93(0.12) C:2.00(0.12)	inspiratory muscle training + CON Length of Intervention:2 weeks Freq: 2 times a day Duration: 40 min	CON	FOIS, FDS	Swallowing function Swallowing safety
Park JS et al.	Korea	2018	T:62.16(17.27) C:58.43(12.51)	T:11/6/5 C:11/4/7	T:4/7 C:5/6	T:6/5/0 C:4/7/0	FDS T:38.64(18.41) C:33.18(16.23)	CTAR exercise + CON Length of Intervention: 4 weeks Freq: 5 times a week Duration: NR	CON	FDS	Swallowing function
Carnaby GD et al.	United States of America	2020	T:70.60(11.80) C:64.30(14.70)	T:18/8/10 C:17/7/10	T:1/17 C:1/16	NR	FOIS T:3.25(1.61) C:4.35(1.80)	exercise-based swallowing therapy + CON Length of Intervention: 3 weeks Freq: daily Duration: 60 min	CON	FOIS, MASA	Swallowing function
Hwang NK et al.	Korea	2019	T:60.50(12.50) C:62.20(10.30)	T:11/6/5 C:10/5/5	T:7/4 C:6/4	T:8/3/0 C:7/3/0	Tongue motility T:2.59(0.86) C:2.49(1.10)	tongue stretching exercises + CON Length of Intervention: 4 weeks Freq: 5 times a week Duration:<10 min	CON	Tongue motility	Swallowing function
Park JS et al.	Korea	2020	T:62.10(10.10) C:61.80(12.10)	T:15/9/6 C:14/8/6	T:8/7 C:6/8	T:6/9/0 C:7/7/0	FOIS T:3.40(0.50) C:3.70(0.50)	RJOE + CON Length of Intervention: 4 weeks Freq: 5 times a week Duration: 30–60 min	CON	FOIS	Swallowing function Swallowing safety

Note: SD, standard deviation; T, experimental group; C, control group; CON, control group with conventional swallowing therapy (no exercise); EMST, expiratory muscle strength training; NMES, neuromuscular electrical stimulation; MIE, mechanical inspiration and expiration; VFSS, videofluoroscopic swallowing study; FOIS, functional oral intake scale; VDS, videofluoroscopic dysphagia scale; MASA, mann assessment of swallowing ability; ASHA-NOMS, American Speech-Language-Hearing Association-National Outcome Measurement System Swallowing scale; FDS, functional dysphagia Scale; EST, effortful swallowing training; TPRT, Tongue-Pressure Resistance Training; TSAT, tongue strength and accuracy training; TPSAT, tongue pressure strength and accuracy training; FCM, functional communication measure for swallowing; CTAR, chin tuck against resistance; HLE, head lift exercise; RJOE, resistive jaw opening exercise.

### 3.3 Risk of bias

**FIGURE 2:**
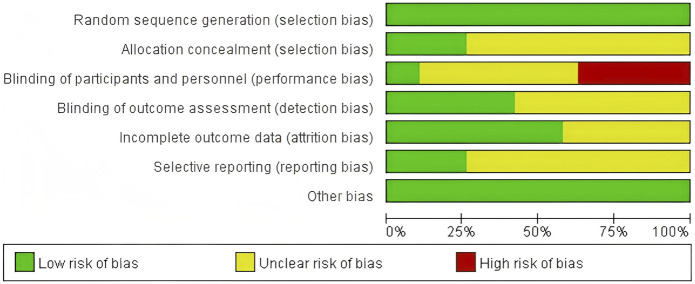
Combined percentage risk of bias in each risk domain for all included trials.

**FIGURE 3:**
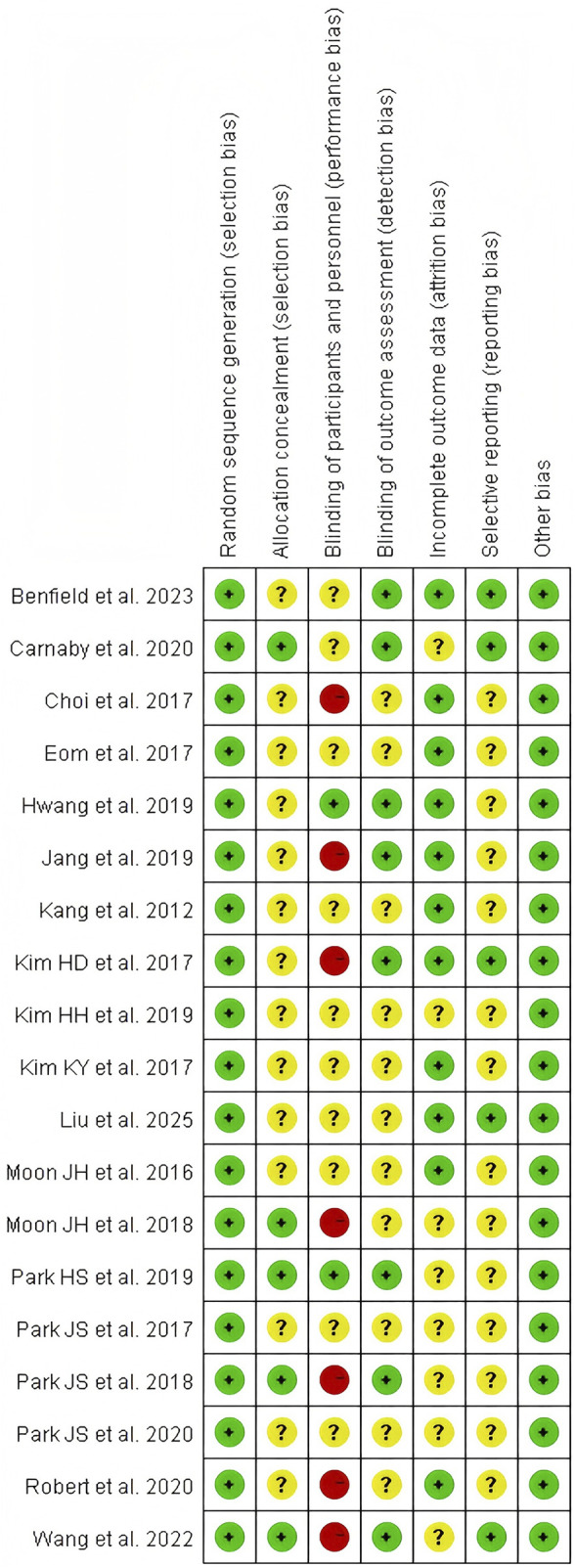
Risk of bias summary: review authors’ judgments about each risk of bias item presented as percentages across all included studies.

Using the Cochrane Bias Risk Assessment Tool for RCTs, all included studies were rated as having a low risk of bias in terms of random sequence generation. Among these 19 studies, five demonstrated a low risk of allocation concealment bias, while the remaining 14 studies posed an unclear risk due to inadequate description of the allocation method. Due to the inherent challenges of implementing double-blinding procedures in resistance exercise interventions, primarily stemming from the visibility of exercise protocols, this domain exhibited the highest risk of potential methodological bias. Only two studies provided explicit descriptions of double-blinding implementation, resulting in an overall high risk of bias for this criterion. Regarding outcome blinding assessment, nine studies ensured that outcome assessors were fully blinded to treatment allocation (i.e., assessor blinding), while 10 studies did not report blinding methods and were thus classified as unclear risk. For incomplete outcome data, 11 studies demonstrated low risk with no participant attrition or dropout, whereas eight studies acknowledged data loss but provided clear explanations for exclusions and data handling, leading to an unclear risk rating. Regarding selective reporting bias, five studies were classified as low-risk in their reporting assessments. However, 14 studies lacked descriptions of pre-registered trial protocols and were categorized as unclear risk. No other sources of bias were identified across all included studies (Figures 2, 3).

### 3.4 Compliance with the ACSM recommendations

Among the 19 included studies, 11 were classified as high ACSM adherence and 8 as low ACSM adherence when applying the 6/8 ACSM adherence criteria. Under the stricter 7/8 ACSM adherence criteria, nine studies met the high adherence threshold, while 10 were categorized as low or uncertain adherence. The lower or uncertain adherence rates were primarily attributed to discrepancies between the exercise dosage (e.g., intensity, frequency, duration) of resistance training protocols and ACSM-recommended prescriptions or insufficient reporting of exercise prescription details for accurate assessment (Table 3).

**TABLE 3:** Resistance exercise evaluated according to the ACSM’s recommendations.

Author, year	Resistance exercise								ACSM adherence
	Frequency		Intensity/workload		Repetitions		Sets		Points (Percent)
	3–5/day week		Start with 40%–50% of 1RM, no more than 60%–80% 1RM		5–10		2–5		
Kang et al. (2012)	7	↓	NR	→	NR	→	NR	→	3/8 (37.5%)
Choi et al. (2017)	5	↑	NR	→	10	↑	3	↑	7/8(87.5%)
Eom et al. (2017)	5	↑	70% 1RM	↑	5	↑	5	↑	8/8 (100%)
Kim et al. (2017)	3	↑	Ind.tail	→	NR	→	NR	→	5/8 (62.5%)
Arnold and Bausek (2020)	7	↓	NR	→	NR	→	3	↑	4/8 (50%)
Jang et al. (2019)	5	↑	Ind.tail	→	NR	→	2	↑	6/8 (75%)
Park et al. (2019)	5	↑	Refer to the ACSM’s Resistance Training Guide	↑	10	↑	3	↑	8/8 (100%)
Kim et al. (2017)	5	↑	60%–80% 1RM	↑	10	↑	3	↑	8/8 (100%)
Moon et al. (2016)	5	↑	50%–100% 1RM	↓	6	↑	5	↑	6/8 (75%)
Moon et al. (2018)	5	↑	75% 1RM	↑	6	↑	5	↑	8/8 (100%)
Wang et al. (2022)	5	↑	Ind.tail	→	NR	→	NR	→	5/8 (62.5%)
Kim and Park (2019)	5	↑	Ind.tail	→	10	↑	3	↑	7/8(87.5%)
Park et al. (2017)	5	↑	70% 1RM	↑	NR	→	3	↑	7/8 (87.5%)
Benfield et al. (2023)	5	↑	Start with 50% of 1RM, ≥70% 1RM	→	NR	→	NR	→	5/8 (62.5%)
Liu et al. (2025)	5	↑	Borg subjective fatigue scale score of 12–14	↑	10	↑	2	↑	8/8 (100%)
Park et al. (2018)	5	↑	Ind.tail	→	30	↓	3	↑	5/8 (62.5%)
Carnaby et al. (2020)	7	↓	Ind.tail	→	NR	→	NR	→	3/8 (37.5%)
Hwang et al. (2019)	5	↑	NR	→	20	↓	NR	→	4/8 (50%)
Park et al. (2020)	5	↑	70% 1RM	↑	NR	→	3	↑	7/8(87.5%)

Note: Ind.tail: individually tailored, NR: not reporte. Up-pointing/green arrow, fulfils recommendation (2 points). Right-pointing/yellow arrow, uncertain fulfilment (1 point). Down-pointing/red arrow, does not fulfil recommendation (0 points).

Based on the directional scoring of swallowing function assessment scales or tools, positively oriented scores were defined as those where a decrease in scale score indicated improvement in swallowing function, and negatively oriented scores as those where an increase suggested functional enhancement. Under the 6/8 ACSM adherence criteria, adherence rates stratified by outcome measures were as follows: For swallowing function (positively oriented scores), five studies demonstrated high ACSM adherence, while two exhibited low or uncertain adherence. For swallowing function (negatively oriented scores), six studies showed high adherence, compared to four with low/uncertain adherence. For swallowing safety, eight studies achieved high adherence, versus three classified as low/uncertain adherence. Under the stricter 7/8 ACSM adherence criteria, the adherence distribution shifted: For swallowing function (positively oriented scores), four studies met high adherence, with three rated low/uncertain. For swallowing function (negatively oriented scores), five studies showed high adherence, balanced by five in the low/uncertain category. For swallowing safety, seven studies maintained high adherence, contrasted with four in the low/uncertain group.

### 3.5 Meta-analysis

#### 3.5.1 Swallowing function (positively oriented scores)

**FIGURE 4:**
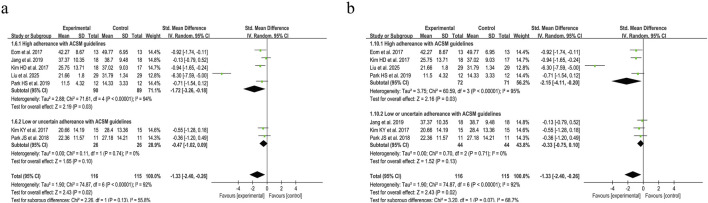
Forest plot analysis of resistance exercise dosage on swallowing function (positively oriented scores) in PSD patients [ACSM compliance classification criteria: **(a)** 6/8; **(b)** 7/8].

**FIGURE 5:**
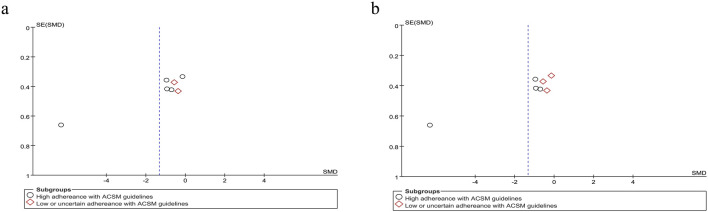
Funnel plot of meta-analysis on the effect of resistance exercise on swallowing function (positively oriented scores) in PSD patients [ACSM compliance classification criteria: **(a)** 6/8; **(b)** 7/8].

Based on the differing scoring directions of swallowing screening measures included in the studies, swallowing function (where positively oriented scores indicate poorer function) was primarily assessed using the VFSS (Kim et al., 2014b), Videofluoroscopic Dysphagia Scale (VDS) (Kim et al., 2014a), and Functional Dysphagia Scale (FDS) (Han et al., 2001). The application of these scales has remained consistent over time. Data from 179 PSD patients across seven studies were included in this study. Given the significant heterogeneity (I^2^ = 92%, p ≤ 0.001), a random-effects model was selected for evaluation. Our analysis revealed a pooled SMD of −1.33 (95% CI −2.40 to −0.26), indicating that resistance training positively impacts swallowing function recovery in PSD patients. Subgroup analysis based on 6/8 ACSM compliance criteria showed that the high-compliance subgroup had an SMD of −1.72 (95% CI -3.26 to −0.18), while the low/uncertain compliance subgroup had an SMD of −0.47 (95% CI −1.02 to 0.09). Similarly, subgroup analysis using 7/8 ACSM compliance criteria demonstrated an SMD of −2.15 (95% CI −4.11 to −0.20) for the high-compliance subgroup, compared with −0.33 (95% CI −0.75 to 0.10) for the low/uncertain compliance subgroup (Figure 4). These findings collectively suggest that strict adherence to ACSM-recommended resistance training demonstrates a more pronounced improvement in swallowing function for PSD patients than those with lower or uncertain compliance. Formal assessment of the funnel plot revealed approximate symmetry between both sides, indicating a lack of publication bias (Figure 5).

#### 3.5.2 Swallowing function (negatively oriented scores)

**FIGURE 6:**
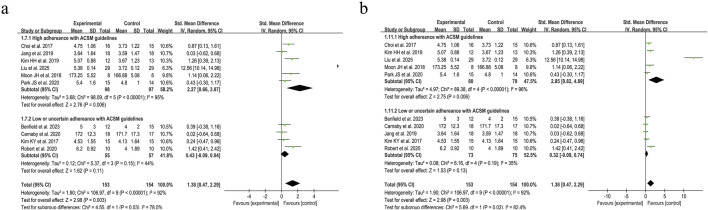
Forest plot analysis of resistance exercise dosage on swallowing function (negatively oriented scores) in PSD patients [ACSM compliance classification criteria: **(a)** 6/8; **(b)** 7/8].

**FIGURE 7:**
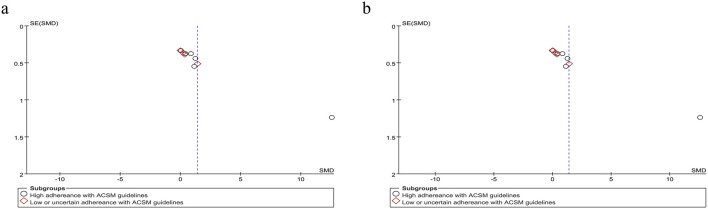
Funnel plot of meta-analysis on the effect of resistance exercise on swallowing function (negatively oriented scores) in PSD patients [ACSM compliance classification criteria: **(a)** 6/8; **(b)** 7/8].

Accordingly, we systematically categorized swallowing screening measures, classifying the Functional Oral Intake Scale (FOIS) (Crary et al., 2005), Mann Assessment of Swallowing Ability (MASA) (Ohira et al., 2023), and American Speech-Language-Hearing Association National Outcome Measurement System Swallowing Scale (ASHA NOMS) (Dungan et al., 2019) as negatively oriented swallowing function assessments, where higher scores indicate greater functional impairment. Moreover, the application of these scales has remained consistent over time. Our analysis encompassed 307 participants across the 10 included studies. Given the substantial heterogeneity (I^2^ = 92%, *p* ≤ 0.001), a random-effects model was applied for statistical analysis. Our analysis yielded a pooled SMD of 1.38 (95% CI 0.47 to 2.29), demonstrating the positive effect of resistance movement on swallowing function recovery in PSD patients. Subgroup analyses based on the 6/8 ACSM adherence criteria revealed a notable disparity between the combined SMD of 2.27 (95% CI 0.66 to 3.87) in the subgroups with high adherence to ACSM and 0.43 (95% CI −0.09 to 0.94) in the subgroups with low or uncertain adherence. To substantiate the robustness of findings, subgroup analyses employing the stringent 7/8 ACSM compliance criteria revealed statistically significant divergence between high-compliance subgroups (SMD = 2.85, 95% CI 0.82 to 4.89) and low-compliance/indeterminate-compliance subgroups (SMD = 0.32, 95% CI −0.09 to 0.74), as detailed in Figure 6. These findings collectively demonstrated that resistance exercise interventions in the high ACSM-adherence subgroup exhibited a superior improvement trajectory in swallowing function. The funnel plot (Figure 7) displayed visual symmetry, and Egger’s regression confirmed no significant asymmetry (*p* = 0.23), validating the robustness of pooled estimates and indicating minimal risk of publication bias.

#### 3.5.3 Swallowing safety

**FIGURE 8:**
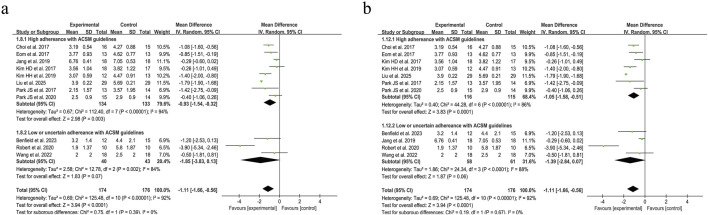
Forest plot analysis of resistance exercise dosage on Swallowing safety in PSD patients [ACSM compliance classification criteria: **(a)** 6/8; **(b)** 7/8].

**FIGURE 9:**
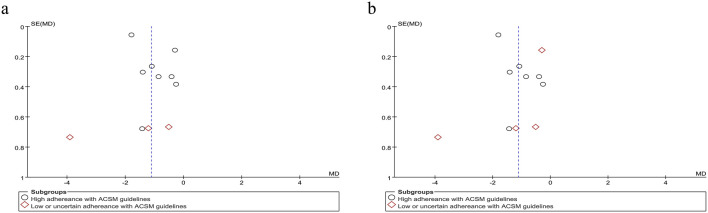
Funnel plot of meta-analysis on the effect of resistance exercise on Swallowing safety in PSD patients [ACSM compliance classification criteria: **(a)** 6/8; **(b)** 7/8].

Swallowing safety in PSD patients was primarily assessed using the Rosenbek Penetration-Aspiration Scale (PAS) (Rosenbek et al., 1996). The PAS accurately identifies patients at risk of swallowing difficulties through its 8-level quantitative criteria, dynamically captures aspiration details, and is widely applicable for the objective assessment of dysphagia caused by various etiologies, such as stroke and neurodegenerative diseases (Burdick et al., 2024). Analysis of 11 trials evaluating swallowing safety outcomes demonstrated considerable between-study heterogeneity (I^2^ = 92%, *p* < 0.001), necessitating implementation of a random-effects model for data synthesis. The pooled SMD of −1.11 (95% CI: −1.66, −0.56) demonstrated a clinically significant improvement in swallowing safety parameters among PSD patients following resistance training interventions. Subgroup analysis under the 6/8 ACSM adherence criteria demonstrated that the high-adherence subgroup (eight studies) had an MD of −0.93 (95% CI −1.54 to −0.32), while the low/uncertain-adherence subgroup (three studies) yielded an MD of −1.85 (95% CI −3.83 to 0.13), with significant between-subgroup differences (*p* < 0.05 for interaction). Under the stricter 7/8 ACSM adherence criteria, the high-adherence subgroup (seven studies) had an MD of −1.05 (95% CI −1.58 to −0.51), and the low/uncertain-adherence subgroup (four studies) exhibited an MD of −1.39 (95% CI −2.84 to 0.07) (Figure 8). Consistently across both thresholds, resistance exercise in high-adherence subgroups demonstrated a favorable impact on swallowing safety. A funnel plot was generated, revealing a symmetrical distribution of effect sizes (Figure 9), with no statistical evidence of publication bias.

## 4 Discussion

This study systematically evaluated 566 PSD patients across 19 included trials, utilizing ACSM adherence as a subgroup stratification factor under the 6/8 ACSM adherence criteria, to comprehensively investigate the impact of resistance exercise dosage on swallowing function and safety in PSD. Furthermore, by refining the ACSM adherence criteria to a stricter 7/8 threshold, we validated the robustness and reproducibility of these findings.

### 4.1 Positive effects of resistance movement on PSD

This meta-analysis demonstrated that resistance exercise significantly improved swallowing function and enhanced swallowing safety in patients with PSD. These findings aligned with current PSD management guidelines and prior research (Vose et al., 2014; Cantemir and Laubert, 2017; Bath et al., 2018), further validating the therapeutic efficacy of resistance exercise as a physical rehabilitation modality for PSD. Current evidence suggests that resistance exercise enhances swallowing biomechanics by training swallowing-related musculature to resist external loads, thereby increasing muscle mass and strength (Burkhead et al., 2007), mitigating neuromuscular degradation, and positively influencing disease reversal (Syed-Abdul, 2021). Notably, different resistance exercise protocols confer benefits at distinct physiological levels: EST improves tongue propulsion forces, directly enhancing bolus transit efficiency (Park et al., 2019). CTAR facilitates safe oral intake progression by optimizing hyolaryngeal complex kinematics, thereby reducing aspiration risk (Park and Hwang, 2021). JOE and Shaker Exercise exhibit comparable activation of suprahyoid muscles (Choi et al., 2020). However, their clinical applicability diverges: The Shaker Exercise enhances anterior cervical musculature strength to improve hyoid displacement and minimize aspiration but may be limited by transient pain and discomfort due to muscle fatigue, potentially compromising patient adherence. In contrast, JOE can be performed in seated positions with reduced physical exertion but imposes higher biomechanical stress on the temporomandibular joint. While resistance exercise demonstrates therapeutic efficacy in swallowing rehabilitation, no single protocol has emerged as universally superior. Accordingly, clinicians should tailor interventions based on patient-specific factors, including musculoskeletal tolerance, aspiration severity, and functional goals.

Emerging evidence indicates that exercise dosage, encompassing intensity, frequency, and duration, is a critical determinant of swallowing function recovery in PSD patients, mediated through exercise-induced neuroplasticity and structural reorganization of cortical swallowing networks (Gauthier et al., 2008; Humbert and German, 2013; Maier et al., 2019). Specifically, increased exercise intensity has been shown to enhance physiological and functional swallowing outcomes, such as pharyngeal constriction velocity and hyoid displacement amplitude (Malandraki et al., 2016). Steele et al.(Steele et al., 2016) further demonstrated that tongue-pressure resistance training directly correlates with improved swallowing safety, effectively reducing thin liquid vallecular residue, a key predictor of aspiration risk. However, excessive exercise dosage may paradoxically diminish therapeutic efficacy by compromising patient adherence due to fatigue or musculoskeletal strain. While resistance exercise is unequivocally beneficial, the optimal dosing parameters (e.g., intensity: %1RM; frequency: sessions/week; volume: sets × repetitions) remain undefined, hindering the development of standardized protocols. This knowledge gap is particularly concerning given the paucity of dose-response studies in PSD populations, as highlighted by our systematic review. Overall, this investigation highlighted the necessity of delineating dose-response dynamics between resistance training parameters (intensity, frequency, repetitions, sets) and functional recovery outcomes in PSD, while establishing an evidence base for precision rehabilitation protocols.

### 4.2 Positive impacts of exercise with high adherence to ACSM guidelines

This study synthesized resistance exercise parameters, including intensity, frequency, repetitions, and sets, from prior research, utilizing ACSM adherence as a subgroup stratification factor to evaluate the impact of varying dosage levels on swallowing function in PSD patients. To our knowledge, this is the first systematic review to employ ACSM adherence criteria for delineating optimal exercise dosages in PSD rehabilitation, addressing a critical gap in evidence-based practice. As the global authority in exercise science, ACSM advocates exercise regimens integrating aerobic activity, resistance training, and flexibility exercises, coupled with regular moderate-intensity physical engagement for health enhancement. Our analysis focused on resistance exercise due to its predominant application in PSD research (Krekeler et al., 2021), driven by the imperative to restore coordinated activation of swallowing-related muscle groups, a physiological demand uniquely suited to resistance modalities in this population. The ACSM explicitly advocates resistance training at intensities sufficient to induce muscle fatigue without exhaustion, emphasizing that adherence to its evidence-based prescriptions benefits even adults with chronic conditions or disabilities (Garber et al., 2011).

To evaluate the efficacy of resistance exercise interventions in PSD, this study incorporated internationally recognized swallowing assessment tools, including VFSS, VDS, FDS, FOIS, MASA, and ASHA NOMS. These tools were systematically selected to comprehensively cover three core dimensions of swallowing function: anatomical integrity, physiological coordination, and functional performance. To ensure clinical interpretability and statistical consistency, outcome measures were classified into positively oriented scales (score reduction indicates functional improvement) and negatively oriented scales (score elevation reflects clinical benefit), strictly adhering to uniform metric definitions throughout the analysis. This standardized classification system facilitated robust data synthesis and enhanced the generalizability of findings. The PAS was prioritized for assessing swallowing safety due to its excellent reliability, strong criterion validity, and direct quantification of penetration-aspiration events, which represents a critical endpoint for PSD risk stratification.

In this systematic review, resistance exercise interventions with high adherence to the 6/8 ACSM criteria demonstrated greater therapeutic benefits in improving swallowing function and safety than those with low or uncertain ACSM adherence. Similarly, under the stricter 7/8 ACSM adherence criteria, high-adherence resistance exercise interventions positively affected both swallowing function and safety. These results confirm the feasibility and clinical utility of ACSM-guided exercise dosages for PSD patients. Current evidence suggests that resistance exercise benefits PSD patients, and this study provides empirical support for implementing ACSM-recommended resistance exercise protocols (including specific dosage parameters) in this population. For clinical application, personalized treatment plans should be developed, with adjustments tailored to individual patient needs while adhering to evidence-based exercise prescription guidelines.

### 4.3 Strengths and limitations of the study

The strengths of this review stem from its standardized methodology and rigorous statistical analyses. To our knowledge, few meta-analyses have hitherto focused on exercise dosage in PSD patients, and none have stratified resistance exercise therapy for PSD based on ACSM adherence criteria. By conducting subgroup analyses based on ACSM adherence levels, this study advances understanding of adherence optimization in tailored resistance exercise prescriptions. Given that resistance exercise constitutes a complex, multifaceted intervention, quantitative evaluation remains challenging. To address this, we implemented a 0–2 scale to systematically assess adherence, minimizing bias arising from incomplete reporting and ensuring stringent inclusion criteria for resistance exercise protocols. Furthermore, to account for the heterogeneity of swallowing function outcomes, we classified outcome measures into positively oriented scores (where lower scores indicate functional improvement) and negatively oriented scores (where higher scores reflect clinical benefit) based on their clinical interpretability. This categorization enhanced the reliability of outcome integration across studies. However, we have to admit that the included studies used a variety of swallowing-related indicators, including VFSS, VDS, and FOIS, and these measures differ to some extent in their evaluation dimensions. Moreover, SMD was used due to the need for statistical synthesis of data from different scales with varying metrics. This method enables the quantitative pooling of results, which is crucial for meta-analytical inference. However, this convenience comes with a trade-off: SMD standardizes effect sizes across different measurement indicators, which may mask the clinical specificity of individual indicators. Additionally, outcome measures were categorized into positively oriented scores and negatively oriented scores, which also increased conceptual heterogeneity to some extent. As highlighted in the Cochrane Handbook, such trade-offs are inherent in synthesizing heterogeneous outcomes, and readers should interpret the combined effects with awareness of the original measures' distinct clinical meanings.

Our study has several limitations that should be acknowledged. First, notable heterogeneity was observed across the included trials due to variations in resistance exercise protocols (e.g., differences in intensity, frequency, and repetitions) and subtle variations in participant characteristics. This lack of standardization complicated the comparison and synthesis of results. A key methodological limitation is the risk of bias inherent in individual studies, primarily attributable to the difficulty in blinding participants/personnel to resistance exercise interventions, which may introduce potential performance and detection bias. As a meta-analysis relying on existing studies, our findings may be influenced by the inherent biases and limitations of the original research. Among these limitations, the heterogeneous characteristics of stroke were not considered, including lesion location, severity, and the presence of cognitive impairment, which may modulate patients’ therapeutic responses to resistance exercise. Stroke-induced dysphagia varies substantially with the neuroanatomical site of injury: for example, lesions involving the brainstem (a critical hub for swallowing reflex integration) are associated with more severe and persistent dysphagia compared to cortical or subcortical lesions. Similarly, larger infarct/hemorrhage volumes or higher stroke severity may reduce patients’ motor learning capacity and treatment adherence, thereby weakening intervention effects. The lack of baseline data in some of the included studies makes it impossible to explore whether these factors modulate the association between ACSM compliance and swallowing outcomes through subgroup analysis. Future research should explicitly record and analyze these variables to optimize patient stratification and refine individualized rehabilitation strategies. In addition, baseline dysphagia severity, an important factor influencing treatment response, was not systematically considered in this analysis. Previous studies have shown that patients with mild dysphagia often exhibit more significant functional improvement with resistance training compared to those with severe dysphagia, due to their preserved neuromuscular reserve and lower risk of aspiration (D'Netto et al., 2023). However, among the 19 included randomized controlled trials, 17 studies reported baseline severity using tools such as VFSS, FOIS, or VDS, but there were significant differences in grading criteria. The remaining two studies did not provide quantitative data on baseline function, merely mentioning the inclusion of “post-stroke dysphagia patients.” This lack of standardized baseline assessment precluded subgroup analyses to explore whether ACSM-recommended protocols yield differential effects across severity strata.

During the data extraction process from figures and tables, despite meticulous efforts to minimize errors, complete avoidance of inaccuracies remains challenging. The inadequate description of exercise prescriptions in some studies led to their classification as not reported (NR), potentially introducing misclassification bias in ACSM compliance assessments. Another notable limitation is the lack of consideration of actual adherence and intervention duration in practice. This study focused on the compliance of resistance exercise prescriptions with ACSM guidelines at the prescription level (e.g., predefined frequency, intensity, and sets) but did not capture real-world execution data, such as the actual number of training sessions completed by patients, deviations from prescribed duration per session, or dropout rates. Previous studies have emphasized that even well-designed exercise protocols may fail to achieve expected efficacy if patients' actual adherence is low (Van Roie et al., 2015). Future studies should systematically record actual compliance indicators (such as training logs and objective monitoring data) within their design to more accurately assess the clinical value of ACSM adherence for PSD. Of course, coexisting oral dysfunction is also a confounding factor worth considering, including poor dentition, reduced tongue strength, and impaired masticatory function, which may independently influence swallowing outcomes. Oral motor skills—such as tongue propulsion, bolus manipulation, and chewing efficiency—are prerequisites for safe swallowing, as they determine the integrity of the bolus before pharyngeal transit. For example, reduced tongue strength impairs bolus clearance from the oral cavity, increasing the risk of residue aspiration; while edentulism or masticatory impairment may lead to insufficient bolus breakdown, complicating swallowing dynamics (Nagashima et al., 2021). Notably, among the 19 included RCTs, only two studies assessed tongue strength as part of their outcomes, with no study systematically recording dentition status or masticatory function. This lack of data precluded evaluation of whether oral dysfunction modifies the association between resistance exercise adherence and swallowing improvement. Future research should incorporate comprehensive oral function assessments to account for this potential source of variability.Finally, the included studies predominantly enrolled middle-aged and older adults (mean age range: 59.26–71.00 years). Consequently, the generalizability of these findings to younger populations is limited.

## 5 Conclusion

Through a systematic review of resistance exercise interventions, this study supports the recommendation that resistance exercise is an effective measure for improving swallowing function in patients with PSD. Furthermore, subgroup analyses under both the 6/8 and 7/8 ACSM adherence criteria consistently demonstrated that resistance exercise interventions with high ACSM adherence yielded significantly greater improvements in swallowing function and safety compared to those with low or uncertain adherence. However, given the limited number of resistance exercise studies in PSD, future research should employ more rigorous experimental designs and larger sample sizes for validation.

## Data Availability

The original contributions presented in the study are included in the article/Supplementary Material, further inquiries can be directed to the corresponding authors.

## Abbreviations

SD, standard deviation; T, experimental group; C, control group; CON, control group with conventional swallowing therapy (no exercise); EMST, Expiratory Muscle Strength Training; NMES, Neuromuscular Electrical Stimulation; MIE, Mechanical inspiration and expiration; VFSS, Videofluoroscopic Swallowing Study; FOIS, Functional Oral Intake Scale; VDS, Videofluoroscopic Dysphagia Scale; MASA, Mann Assessment of Swallowing Ability; ASHA-NOMS, American Speech-Language-Hearing Association-National Outcome Measurement System Swallowing scale; FDS, functional dysphagia Scale; EST, Effortful Swallowing Training; TPRT, Tongue-Pressure Resistance Training; TSAT, Tongue strength and accuracy training; TPSAT, Tongue pressure strength and accuracy training; FCM, Functional Communication Measure for swallowing; CTAR, chin tuck against resistance; HLE, Head Lift Exercise; RJOE, Resistive Jaw Opening Exercise; 1RM, one repetition maximum; Ind.tail, individually tailored; NR, not reported.
