# Supplementary material for: Effects of resistance exercise on patients with post-stroke dysphagia based on ACSM recommendations: a systematic review of randomized controlled trials
Source: Front Physiol. 2025 Aug 6;16:1623298. doi: 10.3389/fphys.2025.1623298 (PMC12364908; doi:10.3389/fphys.2025.1623298)
Supplement: Supplementary file 1 [file Table1.docx]

Supplementary Material

**Effects of resistance exercise on patients with post-stroke dysphagia based on ACSM recommendations: a systematic review of randomized controlled trials**

**Yu Ye^1+^ Kairui Wu^1+^ Yingquan Liu^1^ Hongjie Ji^1^ Hongtao Li^2^ Bo Jiang^1^ Fangyuan Xu^1^ Xuejun Li^3*^ Peijia Hu^3*^ Hongliang Cheng^3*^**

*** Correspondence:**

Hongliang Cheng: chl.75811@163.com;

Peijia Hu:zjyyhpj@163.com;

Xuejun Li:lixuejun0308@126.com

Appendix 1 Search Strategy

| Database | Search strategy | Amount |
| --- | --- | --- |
|  | PUBMED |  |
| #1 | Stroke[MeSH Terms] | 188,490 |
| #2 | (((((((((((((((Stroke[Title/Abstract]) OR (Strokes[Title/Abstract])) OR (Cerebrovascular Accident[Title/Abstract])) OR (Cerebral Stroke[Title/Abstract])) OR (cerebral infarction[Title/Abstract])) OR (Stroke, Cerebral[Title/Abstract])) OR (Cerebrovascular Apoplexy[Title/Abstract])) OR (Brain Vascular Accident[Title/Abstract])) OR (Brain Vascular Accidents[Title/Abstract])) OR (Cerebrovascular Stroke[Title/Abstract])) OR (Stroke, Cerebrovascular[Title/Abstract])) OR (Apoplexy[Title/Abstract])) OR (CVA (Cerebrovascular Accident[Title/Abstract]))) OR (CVAs (Cerebrovascular Accident[Title/Abstract]))) OR (Stroke, Acute[Title/Abstract])) OR (Cerebrovascular Accident, Acute[Title/Abstract]) | 372,923 |
| #3 | #1 OR #2 | 407,051 |
| #4 | Deglutition Disorders[MeSH Terms] | 62,548 |
| #5 | (((((((((Deglutition Disorders[Title/Abstract]) OR (Deglutition Disorder[Title/Abstract])) OR (Disorders, Deglutition[Title/Abstract])) OR (Dysphagia[Title/Abstract])) OR (Swallowing Disorders[Title/Abstract])) OR (Swallowing Disorder[Title/Abstract])) OR (Oropharyngeal Dysphagia[Title/Abstract])) OR (Dysphagia, Oropharyngeal[Title/Abstract])) OR (Esophageal Dysphagia[Title/Abstract])) OR (Dysphagia, Esophageal[Title/Abstract]) | 40,574 |
| #6 | #4 OR #5 | 83,616 |
| #7 | #3 AND #6 | 3,479 |
| #8 | (((post-stroke dysphagia[Title/Abstract]) OR (swallow dysfunction after stroke[Title/Abstract])) OR (dysphagia after stroke[Title/Abstract])) OR (swallowing disorder after dysphagia[Title/Abstract]) | 6,770 |
| #9 | #7 OR #8 | 9,143 |
| #10 | (((Exercise[MeSH Terms]) OR (Resistance Training[MeSH Terms])) OR (Exercise Movement Techniques[MeSH Terms])) OR (Exercise Therapy[MeSH Terms]) | 314,933 |
| #11 | (((((((((((((Exercise[Title/Abstract]) OR (Resistance Training[Title/Abstract])) OR (Exercise Movement Techniques[Title/Abstract])) OR (Exercise Therapy[Title/Abstract])) OR (Exercises[Title/Abstract])) OR (Training[Title/Abstract])) OR (resistance exercise[Title/Abstract])) OR (Physical Exercise[Title/Abstract])) OR (Acute Exercise[Title/Abstract])) OR (Exercise Program[Title/Abstract])) OR (Exercise Training[Title/Abstract])) OR (Physical Activity[Title/Abstract])) OR (Training, Resistance[Title/Abstract])) OR (Strength Training[Title/Abstract]) | 1,063,393 |
| #12 | #10 OR #11 | 1,157,925 |
| #13 | #9 AND #12 | 646 |
| Embase | | |
| #1 | 'stroke:ab,ti OR strokes:ab,ti OR 'cerebrovascular accident':ab,ti OR 'cerebral stroke':ab,ti OR 'cerebral infarction':ab,ti OR 'stroke, cerebral':ab,ti OR 'cerebrovascular apoplexy':ab,ti OR 'brain vascular accident':ab,ti OR 'brain vascular accidents':ab,ti OR 'cerebrovascular stroke':ab,ti OR 'stroke, cerebrovascular':ab,ti OR apoplexy:ab,ti OR (cva:ab,ti AND 'cerebrovascular accident':ab,ti) OR (cvas:ab,ti AND 'cerebrovascular accident':ab,ti) OR 'stroke, acute':ab,ti OR 'cerebrovascular accident, acute':ab,ti | 576,134 |
| #2 | 'deglutition disorders':ab,ti OR 'deglutition disorder':ab,ti OR 'disorders, deglutition':ab,ti OR dysphagia:ab,ti OR 'swallowing disorders':ab,ti OR 'swallowing disorder':ab,ti OR 'oropharyngeal dysphagia':ab,ti OR 'dysphagia, oropharyngeal':ab,ti OR 'esophageal dysphagia':ab,ti OR 'dysphagia, esophageal':ab,ti | 67,666 |
| #3 | #1 AND #2 | 5,240 |
| #4 | 'post-stroke dysphagia':ab,ti OR 'swallow dysfunction after stroke':ab,ti OR 'dysphagia after stroke':ab,ti OR 'swallowing disorder after dysphagia':ab,ti | 721 |
| #5 | #3 OR #4 | 5,242 |
| #6 | 'exercise:ab,ti OR 'resistance training':ab,ti OR 'exercise movement techniques':ab,ti OR 'exercise therapy':ab,ti OR exercises:ab,ti OR training:ab,ti OR 'resistance exercise':ab,ti OR 'physical exercise':ab,ti OR 'acute exercise':ab,ti OR 'exercise program':ab,ti OR 'exercise training':ab,ti OR 'physical activity':ab,ti OR 'training, resistance':ab,ti OR 'strength training':ab,ti | 1,390,364 |
| #7 | #5 AND #6 | 628 |
| Cochrane | | |
| #1 | (Stroke):ti,ab,kw OR (Strokes):ti,ab,kw OR (Cerebrovascular Accident):ti,ab,kw OR (Cerebral Stroke):ti,ab,kw OR (cerebral infarction):ti,ab,kw | 83,399 |
| #2 | (Stroke, Cerebral):ti,ab,kw OR (Cerebrovascular Apoplexy):ti,ab,kw OR (Brain Vascular Accident):ti,ab,kw OR (Brain Vascular Accidents):ti,ab,kw OR (Cerebrovascular Stroke):ti,ab,kw | 26,011 |
| #3 | (Stroke, Cerebrovascular):ti,ab,kw OR (Apoplexy):ti,ab,kw OR (CVA (Cerebrovascular Accident)):ti,ab,kw OR (Stroke, Acute):ti,ab,kw OR (Cerebrovascular Accident, Acute):ti,ab,kw | 36,163 |
| #4 | #1 OR #2 OR #3 | 83,634 |
| #5 | (Deglutition Disorders):ti,ab,kw OR (Deglutition Disorder):ti,ab,kw OR (Disorders, Deglutition):ti,ab,kw OR (Dysphagia):ti,ab,kw OR (Swallowing Disorders):ti,ab,kw | 7,114 |
| #6 | (Swallowing Disorder):ti,ab,kw OR (Oropharyngeal Dysphagia):ti,ab,kw OR (Dysphagia, Oropharyngeal):ti,ab,kw OR (Esophageal Dysphagia):ti,ab,kw OR (Dysphagia, Esophageal):ti,ab,kw | 3,589 |
| #7 | #5 OR #6 | 7,115 |
| #8 | #4 AND #7 | 1,353 |
| #9 | (post-stroke dysphagia):ti,ab,kw OR (swallow dysfunction after stroke):ti,ab,kw OR (dysphagia after stroke):ti,ab,kw OR (swallowing disorder after dysphagia):ti,ab,kw | 1,269 |
| #10 | #8 OR #9 | 1,707 |
| #11 | (Exercise):ti,ab,kw OR (Resistance Training):ti,ab,kw OR (Exercise Movement Techniques):ti,ab,kw OR (Exercise Therapy):ti,ab,kw OR (Exercises):ti,ab,kw | 160,342 |
| #12 | (Training):ti,ab,kw OR (resistance exercise):ti,ab,kw OR (Physical Exercise):ti,ab,kw OR (Acute Exercise):ti,ab,kw OR (Exercise Program):ti,ab,kw | 219,503 |
| #13 | (Exercise Training):ti,ab,kw OR (Physical Activity):ti,ab,kw OR (Training, Resistance):ti,ab,kw OR (Strength Training):ti,ab,kw | 135,026 |
| #14 | #11 OR #12 OR #13 | 299,449 |
| #15 | #10 AND #14 | 594 |
| Web of Science | | |
| #1 | (((((((((((((((TS=(Stroke)) OR TS=(Strokes)) OR TS=(Cerebrovascular Accident)) OR TS=(Cerebral Stroke)) OR TS=(cerebral infarction)) OR TS=(Stroke, Cerebral)) OR TS=(Cerebrovascular Apoplexy)) OR TS=(Brain Vascular Accident)) OR TS=(Brain Vascular Accidents)) OR TS=(Cerebrovascular Stroke)) OR TS=(Stroke, Cerebrovascular)) OR TS=(Apoplexy)) OR TS=(CVA (Cerebrovascular Accident))) OR TS=(CVAs (Cerebrovascular Accident))) OR TS=(Stroke, Acute)) OR TS=(Cerebrovascular Accident, Acute) | 727,544 |
| #2 | (((((((((TS=(Deglutition Disorders)) OR TS=(Deglutition Disorder)) OR TS=(Disorders, Deglutition)) OR TS=(Dysphagia)) OR TS=(Swallowing Disorders)) OR TS=(Swallowing Disorder)) OR TS=(Oropharyngeal Dysphagia)) OR TS=(Dysphagia, Oropharyngeal)) OR TS=(Esophageal Dysphagia)) OR TS=(Dysphagia, Esophageal) | 73,640 |
| #3 | #1 AND #2 | 6,841 |
| #4 | (((TS=(post-stroke dysphagia)) OR TS=(swallow dysfunction after stroke)) OR TS=(dysphagia after stroke)) OR TS=(swallowing disorder after dysphagia) | 6,257 |
| #5 | #3 OR #4 | 10,024 |
| #6 | (((((((((((((TS=(Exercise)) OR TS=(Resistance Training)) OR TS=(Exercise Movement Techniques)) OR TS=(Exercise Therapy)) OR TS=(Exercises)) OR TS=(Training)) OR TS=(resistance exercise)) OR TS=(Physical Exercise)) OR TS=(Acute Exercise)) OR TS=(Exercise Program)) OR TS=(Exercise Training)) OR TS=(Physical Activity)) OR TS=(Training, Resistance)) OR TS=(Strength Training) | 3,393,552 |
| #7 | #5 AND #6 | [1,548](https://webofscience.clarivate.cn/wos/alldb/summary/07b6e024-3d69-42cb-b0e2-835057db53e1-01489761c5/relevance/1) |
